# To Imitate Breathing

**Published:** 1879-08

**Authors:** 


*To imitate Breathing.*—Standing at the patient's head, grasp the arms just above the elbows, and draw the arms gently and steadily upward above the head, *and keep them stretched* upward for two seconds. *Thus air is drawn into the lungs.* Then turn down the patient's arms, and press them gently and firmly for two seconds against the sides of the chest. *Thus air is pressed out of the lungs.* Repeat these measures about fifteen times a minute till an effort to respire is perceived; then cease to imitate the movements of breathing and proceed to *induce circulation and warmth* by rubbing the limbs upward with firm grasping pressure and energy, using handkerchiefs, flannels, etc., in order to propel the blood toward the heart. The friction must be continued under the blanket or over the dry clothing.

Promote warmth of the body by the application of hot flannels, bottles or bladders of hot water, heated bricks, etc., to the pit of the stomach, the arm-pits, between the thighs, and to the soles of the feet.

On the restoration of life, a teaspoonful of warm water should be given ; and then if the power of swallowing has returned, small quantities of wine, warm brandy and water, or coffee should be given. The patient should be put to bed and sleep encouraged.

